# Personalized Neonatal Therapy: Application of Magistral Formulas in Therapeutic Orphan Populations

**DOI:** 10.3390/pharmaceutics17080963

**Published:** 2025-07-25

**Authors:** Wenwen Shao, Angela Gomez, Alejandra Alejano, Teresa Gil, María Cristina Benéitez

**Affiliations:** 1 Facultad de Ciencias de la Salud, Universidad Rey Juan Carlos, 28922 Alcorcón, Spain; 2 Área de Farmacia y Tecnología Farmacéutica, Departamento de Ciencias Básicas de la Salud, Universidad Rey Juan Carlos, 28922 Alcorcón, Spain

**Keywords:** newborn, extemporaneous preparation, unlicensed drug, drug preparation, personalized medicine

## Abstract

This review explores the potential of magistral formulas (MFs) as a viable option to meet the needs of neonates, given the lack of adequate therapies for this vulnerable group. The scientific literature on medicines available for neonates is limited. The physiological differences between neonates and adults make it difficult to formulate these medicines. In addition, there are a variety of difficulties in conducting research on neonates: few clinical trials are performed, and there is frequent use of unauthorized medicines. Pharmacokinetics in neonates was investigated in comparison to adults, and different aspects of the absorption, distribution, metabolism, and excretion were observed. One of the main problems is the different pharmacokinetics between the two populations. It is necessary to promote and allow research related to pediatric drug design, approve a specific authorization for use in age-appropriate dosage forms, and improve the quality and availability of information on drugs. This study focused on the MFs typically used for pediatrics, specifically for neonates, analyzing the pharmaceutical forms currently available and the presence of indications and dosage recommendations of the European Medicines Agency. Medications were classified according to therapeutic group, as antihypertensives, corticosteroids, and antiepileptics. The use of off-label medicines remains high in neonatal intensive care units and in primary healthcare, besides in the preparation of MFs by pharmacists. The shortage of medicines specifically designed and approved for neonates is a serious problem for society. Neonates continue to be treated, on numerous occasions, with off-label medicines. Studies and research should be expanded in this vulnerable population group.

## 1. Introduction

The neonatal population presents unique physiological and pharmaceutical characteristics that complicate pharmacological treatments. In addition, most of the drugs currently on the market lack specific indications and dosage recommendations for neonates. This situation leads to frequent use of treatments with off-label indications in this population, which makes it difficult to guarantee safety and efficacy in their clinical management. In response to this problem, the present work aims to investigate and evaluate the therapeutic options available for neonates.

The peculiar characteristics of neonatal pharmacokinetics show that this population exhibits rapid and significant development and maturation, involving changes in body composition, metabolic enzyme activity, fluid distribution, and hepatic and renal function. To meet the unique needs of these changes, specialized formulations are required. Factors such as increased volume of distribution, reduced biotransformation metabolism, and reduced clearance contribute to prolonging the half-life of drugs in neonates.

There are several unresolved clinical challenges due to the shortage of specific medications for this group. Sometimes, medications for adults are used, adjusting the dose, which does not guarantee either efficacy or safety since specific clinical studies are lacking, and errors can occur. Since there are few alternatives for NBs, MFs can be used, although further studies are necessary. The market for neonatal medications is very small, which discourages the pharmaceutical industry from investing in the development of new medications.

The existence of scientific gaps in pharmacotherapy for NBs has been verified, and this poses a serious problem since it affects the safety and efficacy of treatment. The utilization of unlicensed drugs prepared in hospital pharmacies and off-label medication use can lead to problems of ineffectiveness and, even worse, toxicity for this delicate population.

The following materials and methods have been used to address this issue.

Bibliographic searches were performed, consulting a wide variety of sources, including books, research articles, and academic theses. The sources used were PUBMED, GOOGLE SCHOLAR, and the libraries of universities such as URJC. In addition, Artificial Intelligence, such as Copilot and DeepL, has been used.

For the literature search, a series of keywords relevant to the topic of this study were used, such as “newborn”, “drug”, “formulary”, “off-label”, “drug compounding”, “pediatric formulations”, and “extemporaneous magistral”. To improve the effectiveness of the search, Boolean operators such as “AND” and “OR” were used to combine these keywords.

The exclusion criteria were opinion articles, reflection articles, and conference proceedings. The inclusion criteria were summaries of product characteristics, articles in scientific databases, and articles in the field of pharmaceutical technology and clinical pharmacy, including hospital settings.

## 2. Neonatal Pharmacotherapy: Fundamental Concepts

### 2.1. Pediatric Population

The pediatric population can be subcategorized according to age (Table 1) [1].

Newborns (NBs) are considered healthy or full-term NBs when they are born after the 37th week of gestational age [2,3,4]. From a healthcare perspective, the neonatal period encompasses the first 28 full days of life in full-term NBs [5].

An NB is considered premature if it is born alive before completing 37 weeks of gestation. It can be classified into subcategories according to gestational age: extreme preterm (less than 28 weeks), very preterm (28 to 32 weeks), and moderate to late preterm (32 to 37 weeks) [6]. The neonatal period for preterm NBs is from birth to the expected date of delivery plus 27 days [1].

According to the World Health Organization (WHO), low birth weight is defined as being less than 2500 g. This problem continues to be a worldwide public health concern, associated with high economic costs and various short- and long-term consequences. Approximately 15% to 20% of newborns in the world have low birth weight, equivalent to more than 20 million newborns annually [7].

In 2022, low birth weight NBs accounted for 6.61% of all births in Spain [8]. However, they account for the largest proportion of neonatal deaths. Low birth weight NBs are more likely to die in the neonatal period than term NBs. This probability is even higher in very low birth weight NBs [9]. These data indicate that low or very low birth weight NBs require intensive care and special attention to increase survival rates (Table 2) [9].

### 2.2. Principles of Pharmacotherapy in Neonates

To ensure safe and effective treatment in neonates, it is necessary to understand the pharmacokinetic principles and physiological characteristics of this population group [10]. Pharmacokinetics encompasses processes such as drug absorption, distribution, metabolism, and excretion, which are influenced by changes in body composition and organ functions throughout life [11]. Although pediatric patients are often classified according to postnatal age for drug administration, it is important to recognize that these changes do not follow a linear relationship with age, being most dramatic during the first 18 months of life, with dynamic changes in organ functions and the need for special therapeutic guidelines.

Apart from age, pharmacokinetics can also be influenced by intrinsic (gender, genotype, ethnicity, and hereditary diseases) and extrinsic (acquired diseases, xenobiotic exposure, and diet) factors during the first two decades of life [12,13].

**Figure 1:**
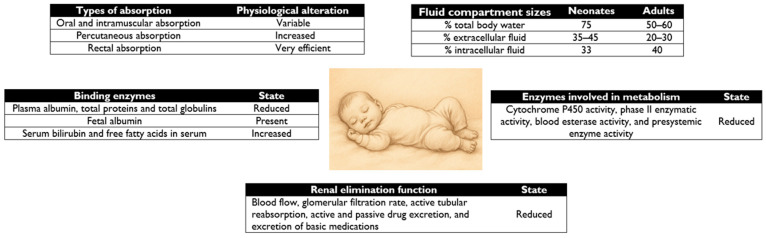
Comparison of neonatal pharmacokinetics versus adult pharmacokinetics.

The different pharmacokinetic characteristics of NBs are detailed in Figure 1.

#### 2.2.1. Drug Absorption

The absorption of orally administered drugs is influenced by physiological factors in the stomach, intestines, and bile ducts. pH, passive diffusion, and gastrointestinal motility primarily affect the rate and extent of drug absorption.

In NBs, a variation in gastric pH is observed in the first hours of life (pH 6–8), with an initial decrease (pH 2–3) and then an increase in pH due to immature parietal cells [13]. This change affects the ionization of drugs, which increases the absorption of weakly basic drugs (such as penicillin G, amoxicillin, nafcillin, and erythromycin) and decreases the absorption of acidic drugs (such as phenobarbital and phenytoin) [14]. In addition, neonates have irregular gastric emptying time and reduced intestinal motility, which affects active ingredients with reduced water solubility (such as phenytoin and carbamazepine) [13]. Conversely, NBs, and in particular preterm infants, have reduced bile acid reserve and biliary function, resulting in reduced solubilization and absorption of lipophilic drugs and fat-soluble vitamins [13,14]. These characteristics are summarized in Table 3, which compares gastrointestinal absorption in neonates with normal values in the adult population.

The safest and most accurate way to administer drugs is intravenously, as it ensures full bioavailability. However, neonatal development can also affect the absorption of drugs from tissues and organs.

Intramuscular absorption in neonates is adversely affected by the higher percentage of water per unit muscle mass, reduced muscle mass, perfusion, and contractility, and changes in intramuscular blood flow. This makes absorption by this route variable and unpredictable [15,16].

Rectal absorption in this population may be higher, but bioavailability is less predictable due to variations in depth of administration or drug retention [14,15,17,18].

Transdermal absorption in neonates is increased because they have a very thin stratum corneum and a more hydrated epidermis, a higher body surface area to body weight ratio [11], all of which can increase the risk of toxicity from drugs applied to the skin [13]. NBs younger than 2 weeks should avoid this route [19,20].

#### 2.2.2. Distribution of Drugs

The distribution of a drug in the body is influenced not only by its physicochemical properties, such as molecular weight, degree of ionization, and partition coefficient, but also by age-related changes in body composition, the amount of plasma proteins, and their ability to bind to the drug [13,15].

The volume of distribution is determined according to the ratio of total body water/extracellular water, fat, and protein binding. These factors vary in neonates and adults, affecting drug concentration in plasma and tissues.

In neonates, the percentage of total body water and extracellular water is higher than in adults [11] and, as shown in Figure 1, leads to an increase in the volume of distribution of water-soluble drugs with low affinity for proteins, such as sulfonamides, penicillins, and aminoglycosides [15,21].

Neonates have a lower proportion of body fat (12%) compared to adults (18%), which reduces the absorption of fat-soluble drugs. Despite this, the lipid content in the developing central nervous system is high. The blood–brain barrier (BBB) is not fully developed, which may increase the distribution of lipophilic drugs and thus their central effects [14,19,20].

Age is a determining factor in the protein binding of drugs, affecting protein concentrations and/or their binding affinity [13]. In neonates, there is a higher free fraction of drugs due to the lower concentration of binding proteins such as albumin (affecting the distribution of ampicillin, penicillin, cloxacillin, diazepam, and flucloxacillin), alpha 1-glycoproteins (affecting alprenolol and lidocaine), and lipoproteins. In addition, these proteins have lower binding capacity.

In any case, neonates may present higher levels of fatty acids and bilirubin that compete with the drug for binding to albumin [15,22].

#### 2.2.3. Drug Metabolism

Drug elimination through biotransformation is slower in neonates than in adults [23]. Although the liver plays a key role in drug metabolism, other organs such as the kidneys, small intestine, lungs, adrenal glands, blood, and skin may also be involved in the biotransformation of certain compounds [13].

On the other hand, liver function in neonates is deficient, with lower activity of oxidative enzyme systems [11]. Drugs that are metabolized by the hepatic route have a slower clearance and a longer elimination time than in adults, because the phase I and phase II metabolization systems are not fully developed. Examples of affected drugs include local anesthetics [13,24].

#### 2.2.4. Drug Excretion

In the elimination of drugs and their metabolites from the body, the kidneys play an important role. However, in neonates, decreased renal function is observed in proportion to their body weight and body surface area. This phenomenon is due to a reduction in renal blood flow, as well as a decrease in glomerular filtration rate (GFR) and renal tubular activity for drug reabsorption and secretion. This is due to the anatomical and functional immaturity of the kidneys [14,25].

In term neonates, the estimated GFR represents approximately 30–40% of the values observed in adults [26]. Although they have a sufficient number of glomeruli, the GFR is limited by the smaller filtration surface of the glomerular capillary [27]. The GFR at birth averages 10–20 mL/min/1.73 m^2^ and doubles during the first two weeks of life due to decreased renovascular resistance and increased renal blood flow. At 10–12 months, the GFR approaches adult values [11,13,15,28].

Adjustments in the dosing interval may be required to avoid complications. Caution should be exercised with the use of drugs with a narrow therapeutic index in neonates, such as aminoglycosides, digoxin, and vancomycin [20].

### 2.3. Newborns: A Special Group

Because NBs are small in size and unstable in weight, it can be difficult to determine the appropriate standard therapeutic dose. Dosage can be calculated using body weight or body surface area. When preparing formulations for NBs, pharmaceutical collaboration is necessary, as NBs often receive very small doses [29].

Pediatric pharmaceutical research presents numerous challenges, which are intensified when dealing with the neonatal population. Drug research tends to focus on the adult population, which represents the largest and most profitable market. As a result, research in other population groups, such as pediatrics in general, remains a low priority and is often never conducted. The following are among the main difficulties for clinical research in pediatrics [29,30,31]:

- Methodological requirements: recruitment of pediatric population for research, pharmacokinetic characteristics different from adults, lack of adequate formulations, insufficient specific indications, and lack of incentives for research.
- Ethical problems: clinical trials in children face significant ethical dilemmas, and there is distrust on the part of parents.
- High costs and limited market: the development of pediatric drugs involves high costs and a small and fragmented market, which reduces the return on investment for laboratories.

In view of this situation, several measures have been taken to address these problems and improve the accessibility of safe and effective drugs in neonates [31].

#### 2.3.1. Adaptation of Dosage Forms and Routes of Administration

The study of pharmaceutical forms is designed to meet the specific dosing needs of neonates, allowing a more precise and safer administration.

##### Parenteral Route

Parenteral administration is widely used in neonatology, especially intravenous administration in preterm or critically ill NBs. Venous access in NBs is provided by several types of catheters, including peripheral catheters, umbilical catheters, and peripheral catheters inserted into the central vein, as shown in Table 4 [29].

An important factor to consider is the amount of intravenous fluid administered. NBs tolerate about 100–140 mL/kg/day. This means an injection volume of 10–20 mL/h [29,32].

If the drug needs to be diluted during use, excipient solutions such as 5% and 10% (*w*/*v*) dextrose and 0.9% (*w*/*v*) sodium chloride should be used to maintain isotonicity. Dextrose can also restrict sodium and serve as an energy source, as immature renal function in neonates alters the ability of the kidneys to filter sodium and other electrolytes efficiently. It is important to avoid using water for IV injection formulations, as it can create a hypotonic solution [29].

Because RNs have limited intravenous access, intravenous medications or parenteral nutrition are often administered through the same catheter [33]. This can reduce the speed of administration, prolong exposure, increase drug interactions, and increase the risk of incomplete dissolution or precipitation due to small volumes of solution [29,34]. They may also form precipitations due to incompatibilities caused by different physicochemical properties of the injected solution [35].

##### Oral Route

A major concern when administering medications to NBs is their ability to swallow effectively; this problem is encountered even in the administration of syrups [29]. According to the EMA guideline [36], oral formulations such as powders and granules for dissolving by measuring devices and liquid dosage forms for oral administration (solutions, suspensions, and oral drops) are suitable for use in NBs. The maximum recommended volume of a single dose may be as little as 0.1 mL.

Dosing devices such as oral syringes or droppers have accuracy issues [37]. New designs are being investigated, such as a therapeutic nipple shield, which involves placing the shield on the drug-loaded nipple on the mother’s breast during breastfeeding [38] and home drug-dispensing pacifiers, which consist of a syringe attached to a pacifier, designed to push the drug through the pacifier to avoid the infant’s taste buds and reduce the difficulty of liquid drug administration [39,40,41].

Oral formulations are the most cost-effective and present less therapeutic risk. In addition, drug dispensers aid adherence and facilitate administration. However, these preparations have problems of stability and palatability (in terms of texture and taste), and can interact with tubes, powdered milk replacers, and other medications [42].

##### Rectal Route

Rectal absorption of drugs in NBs is unpredictable, as fecal incontinence or drug retention capacity must be taken into account [29].

This route is considered when intravenous or oral administration is not possible. It is supported by a study in very low birth weight preterm NBs comparing rectal ibuprofen and oral ibuprofen for closure of hemodynamically significant patent ductus arteriosus. This study demonstrates similar efficacy for both routes [43].

##### Pulmonary Route

Inhaled administration in neonates shows uncontrolled absorption and increased systemic effects, which increases the risk of toxicity. Therefore, efficacy with this route is also difficult to predict [14].

Although the pulmonary route offers the advantage of fewer side effects [44], it is not widely used in neonates, being employed in cases such as surfactant administration [29,45]. It is also used for drugs such as beta agonists, steroids, ribavirin, and sometimes adrenaline [29].

Formulations for nebulization must take into account the solubility and stability of the active substance. However, only a few excipients are approved for inhalation use, making proper formulation difficult [46]. In addition, aerosolized drug deposition in the lungs may vary according to the respiratory rate and respiratory characteristics of the patient, making it difficult to predict the dose received by neonates [29].

##### Nasal Route

The small diameter of the nasal passages and the delicate mucous membranes, often covered with mucus, provide an alternative route for local administration of the drug, achieving a systemic effect, which reduces the need for injections [29].

It is less invasive than the IV route. Compared with the oral route, it has a faster effect and greater bioavailability because it passes directly into the bloodstream, avoiding the first step. However, it has drawbacks compared with other routes, as it has higher permeability to the BBB, which may result in higher toxicity if central effects are not sought.

During the development of intranasal formulations, factors such as the ideal volume per nostril (0.1 mL in neonates), the need for accurate devices to dose small volumes, and the choice of excipients should be considered. For example, penetration enhancers by this route may cause irritation, and many excipients do not have safety data for neonates [29].

##### Transdermal Route

An NB’s skin barrier is not yet fully formed, the type and proportion of lipids are changing, and skin appendages, such as sweat glands and hair follicles, are still forming [47]. In addition, common conditions such as diaper rash and eczema/dermatitis can also make the skin barrier more sensitive [29].

Body surface area (BSA) is an important parameter for drug dose calculation. The most universal and easy-to-use formula for children of all age categories is BSA (m^2^) = square root of [height (cm) × weight (kg)/3600] [48].

As a limitation of the formula, note that height measurement in NBs is difficult and associated with low accuracy [49]. Moreover, NBs have a high surface area to body weight ratio, which affects the calculation of BSA levels and thus increases the risk of unnecessary systemic absorption.

Formulation should ensure the topical and systemic safety of the active ingredients, use excipients with low irritation power, and ensure adequate pH and tonicity. Furthermore, taking into account the delicate skin of NBs, the composition should be liquid and easy to apply [29].

#### 2.3.2. Promoting the Availability of Pediatric Formulations

At the European level, the European Regulation (EC) 1901/2006 on pediatric drugs has been implemented [50]. It has three main objectives:

- Promote and allow research related to pediatric drug design.
- The majority of drugs used in the pediatric population should have specific authorization for use in age-appropriate dosage forms.
- Improve the quality and availability of information on drugs used in the pediatric population.

To meet these objectives, several measures have been taken:

- Creation of the Pediatric Committee: A committee established by the European Medicines Agency (EMA) that provides advice on issues related to medicines for pediatric use, and performs drug assessment and approval of pediatric investigation plans.
- Pediatric Investigation Plan: This must demonstrate the quality, efficacy, and safety of medicines.
- European Database of Clinical Trials in Pediatrics: This allows access to information on pediatric clinical trials.
- Reward System: Includes a 6-month patent extension and 15 years of validity for certain drugs.
- Free Scientific Advisory Service.

In 2017, the EMA Pediatric Committee presented a report on ten years of experience gained since the implementation of this pediatric regulation [51]. According to the report, between the years 2007 and 2016, a 50% increase in the proportion of clinical trials registered in the European EudraCT database incorporating minor participants was observed. This proportion increased from 8.25% to 12.4% during that period.

## 3. Analysis of Magistral Formulas in Neonatal Therapeutics

In European countries such as Spain, there are specific regulations that deal with magistral formulas (MFs). An MF is defined as a drug intended for an individualized patient, prepared by the pharmacist, or under his or her direction, to expressly fulfill a detailed medical prescription of the medicinal substances it includes, according to the technical and scientific standards of the pharmaceutical art, dispensed in his/her pharmacy or pharmaceutical service and with due information provided to the user [52].

There is also the National Formulary (NF) [53], which is an official publication that compiles typical MFs and the official preparations recognized as drugs. This document details the categories, indications, and raw materials used in their composition and preparation, in addition to establishing the regulations for their correct preparation and control, as stipulated in article 44 of Law 29/2006, of 26 July [54]. The monitoring of MFs makes it possible to guarantee the quality, safety, and effectiveness of the drugs produced.

Pediatric formulations in a European market, such as Spain, showed that the vast majority of pediatric formulations are available in the form of oral liquid formulations. Liquid presentations allow for more accurate dosing but have limitations, such as a shorter shelf life and the need for specific storage conditions, for example, refrigeration [31].

The following is an analysis of some of the pediatric-type MF monographs, classified according to their therapeutic use.

### 3.1. Antihypertensives

Defining arterial hypertension (AHT) in NBs is complicated because of the rapid variations in blood pressure in the first days and weeks of life, especially in premature infants. Blood pressure in neonates depends on birth weight and gestational age. During the first month of life, blood pressure can experience an increase of more than 20% [55], considering AHT of 105–110/65–70 mmHg; although these normative data are still weak, a standard needs to be established [56].

In 2022, a review of the use of formulations for cardiovascular treatments in neonatology indicates that the most indicated cardiovascular drugs in neonatology are hydrochlorothiazide, furosemide, hydralazine, enalapril, captopril, nifedipine, propranolol, spironolactone, and carvedilol [42]. Only some of them, including captopril, furosemide, and hydrochlorothiazide, are available in the NF.

The following are the MFs typed for neonates in the NF:

- Captopril 1 mg/mL oral solution

Captopril is one of the few antihypertensives that have been studied in NBs. It has been shown to lower blood pressure effectively but may cause a sudden drop in blood pressure, especially in neonates [57,58].

Within the EMA database, information is only available on pharmacovigilance reports making references to drugs authorized by other types of procedures, such as the national one. In the Spanish national CIMA database, the authorized and marketed drugs containing captopril as the sole active ingredient are only available in tablet form. The indications and dosage recommendations for the pediatric population, including neonates, are detailed in the technical data sheet of the drugs currently available on the Spanish market.

Although this data sheet provides detailed dosing recommendations for neonates, the pharmaceutical forms available are not adequate. Therefore, as in previous antihypertensives, the use of the NF should be resorted to since it has MFs with pharmaceutical forms suitable for this type of population [59].

Importantly, the administration of captopril should be initiated under close medical supervision, since neonates are more vulnerable to hemodynamic adverse effects [60].

- Enalapril maleate 1 mg/mL oral solution

Enalapril is a drug of the angiotensin-converting enzyme inhibitor (ACEI) group. It is mainly used to treat AHT, heart failure, and proteinuria nephrotic syndrome [61].

Within the EMA database, the authorized and marketed medicines containing enalapril as the sole active ingredient only exist in the form of orodispersible tablets. The indications and dosage recommendations for children from birth to 18 years of age are detailed in the data sheet [62].

For administration, it should be placed on the tongue or in the oral cavity and allowed to disperse.

As in the previous case, although the technical data sheet of the drugs currently available in the Spanish market details dosing recommendations for children from birth, the pharmaceutical forms available are not suitable for this type of population since the size/shape of the drug can lead to a dosing error.

The NF details the indications and dosing recommendations for neonates and the pediatric population [63].

### 3.2. Corticosteroids

Corticosteroids are a group of drugs with potent anti-inflammatory action. Their use in neonatology is limited due to their various adverse effects, such as growth retardation, adrenal suppression, and even alterations in brain development [64].

Although caution should be exercised when using it, there are studies of its use in the prevention or treatment of bronchopulmonary dysplasia in premature neonates [65].

- Dexamethasone 1 mg/mL oral solution

Dexamethasone is a synthetic glucocorticoid drug that has strong anti-inflammatory and immunosuppressive properties [61].

Within the EMA database, authorized and marketed medicinal products containing dexamethasone as the sole active substance are found as tablets [66].

There is no information available for neonates in the NF, which only mentions that it is indicated in adults.

The NF details the indications and dosing recommendations for the pediatric population, but there is no information available for neonates [67].

- Hydrocortisone 1 mg/mL oral suspension

Hydrocortisone is a glucocorticoid for systemic use [61].

Within the EMA database, the authorized and marketed medicinal products containing hydrocortisone as the sole active substance are in the form of modified-release hard capsules. The label details the indications and dosage recommendations only for adolescents from 12 years of age and adults [68].

The NF details the indications and dosing recommendations for the pediatric population, but there is no information available for neonates [69].

### 3.3. Antiepileptics

Gabapentin is the only antiepileptic drug described in the NF. However, a Food and Drug Administration (FDA) report on gabapentin indicates the presence of worrying results in preclinical studies and labels it as possibly “developmentally harmful”, with an undetermined risk to the developing fetus and neonate [70].

- Gabapentin 50 mg/mL oral solution

Gabapentin is an antiepileptic indicated for epilepsy and peripheral neuropathic pain [60,61].

Within the database of European countries such as Spain (CIMA), the authorized and marketed drugs containing gabapentin as the sole active ingredient are in the pharmaceutical forms of hard capsules and coated tablets. The indications and dosage recommendations for the pediatric population aged 6 years and older are detailed in the technical data sheet.

In this case, the NF mentions the indications and dosage recommendations for the pediatric population aged 6 years and older but does not have information for neonates [71].

## 4. Clinical Use and Critical Analysis

### 4.1. Clinical Use

#### 4.1.1. Use of Off-Label and Unlicensed Medicines in a Neonatal Intensive Care Unit

In 1998, Turner defined the term off-target use (OTC), which describes the practice of using drugs in a manner that differs from the specifications set out in their technical instructions for use (TI) or in their marketing authorization. This may result in variations in the prescribed dose, the therapeutic indication, the age of the patient to whom it is administered, and the route of administration. This category also includes unlicensed (UL) drugs that comprise those drugs that do not have an official marketing authorization and can be prepared as magistral formulas [72,73].

Since there is little information from clinical trials specifically aimed at children, pediatricians are forced to rely heavily on the results obtained in studies conducted in adults. This means that, when prescribing medications, pediatricians rely on their own experience and extrapolation, rather than on results with solid clinical research [72,74]. To overcome the problems associated with calculating doses according to body weight, formulations of standard concentrations [75], specifically for alert medications (e.g., dopamine, dobutamine), have been implemented in neonatal intensive care unit settings [29,76,77].

In an observational, descriptive, and retrospective study on the use of OTC and UL drugs in a Spanish neonatal intensive care unit over a three-month period [72], medical prescriptions of three categories were evaluated: prescriptions according to the technical data sheet, OTC prescriptions, and UL prescriptions. As a result, they obtained 54% TI prescription, 41% OTC, and 5% UL. More than 90% of neonates received at least one OTC treatment, with a mean of 3 OTC prescriptions per patient. The most commonly used OTC drugs were ampicillin (due to patient age), gentamicin (due to dosing frequency), and caffeine citrate formulations (due to greater efficiency, despite the existence of a commercial form).

#### 4.1.2. Use of Off-Label Medicines in Primary Care in European Countries Such as Spain

A five-year (2015–2019) observational study [78] on off-label prescribing of drugs in NBs and infants up to 1 year of age in primary care in Spain obtained the following results: during the five years, on average, 34.50% of all prescriptions for 0–1 years were OTCs, of which 17.93% were not based on clinical evidence. Posology data were not available in 88% of the cases. Nearly 13% of the OTC drugs were OTCs. In a 2015–2016 subanalysis, 47.46% of neonatal prescriptions were OTCs.

The most prescribed OTC medications in primary care are salbutamol, topical tobramycin, and cholecalciferol, with more frequent use in the NB and infant groups.

Furthermore, this study again affirms the lack of therapeutic indications in the neonatal population.

### 4.2. Critical Analysis

This work has addressed certain aspects of neonatal pharmacotherapy, highlighting the need to develop and adapt specific treatments for this population.

Drug formulation for neonates requires consideration of factors such as unpredictable absorption, tolerance, compatibility, and physicochemical stability. The need for individualized dosing based on body weight or body surface area highlights the importance of proper drug preparation and administration in NBs.

The adaptation of pharmaceutical forms and routes of administration are important factors in neonatal care. The parenteral route presents several difficulties at the site of administration, while liquid dosage forms for oral administration, although easy to administer and to adjust dose, have problems of pharmacokinetic variability in the absorption of drugs in the gastrointestinal tract.

We investigated the commercialized treatments available for pharmacological treatment in neonates, revealing a scarcity of information on the use of drugs in this population.

The analysis of the magistral formulas for pediatrics described in the regulations of European countries (National Formulary) reveals a scarcity of detailed information on the indication and dosage for neonates. Although some drugs, such as captopril, include dosing recommendations for neonates, approximately half of the formulations (9/20) lack sufficient data.

According to the latest available data, the high percentage of off-label medicines used in neonatal intensive care units and primary care in Spain is noteworthy, reflecting the lack of approved treatment options and the need for adapted therapies in this population.

## 5. Conclusions

- Specialized formulations are presented as a viable solution for personalized, precise dosing tailored to the specific needs of neonates.
- The importance of understanding the pharmacokinetics and physiological characteristics of neonates to improve the efficacy and safety of treatments is highlighted. Although the field of pharmacokinetics has been studied in recent decades, further research is essential to improve and promote specialized formulations.
- The research and development of new dosage forms that offer ease of administration and accuracy in dosing are points to investigate to improve access and efficacy of treatments.
- Despite advances in pediatric research, neonates remain “therapeutic orphans”. To solve this problem, there is a European regulation to promote research in pediatrics that details, among other aspects, promoting and allowing the design of pediatric drugs.

Community pharmacies and hospital pharmacies play an essential role in the development of magistral formulas. Therefore, both pediatricians and pharmacists must be well trained on the specific dosage and formulation needs to provide optimal treatment for neonates.

## Figures and Tables

**Table 1:** Postnatal pediatric age classification.

Category	Postnatal Age
Premature newborn	Born before completing the 37th week of gestation, from birth to the expected date of delivery 27 days
Term neonates	0–28 days
Infants	28 days–23 months
Children	2–11 years
Teenagers	11–18 years

**Table 2:** Relationship between birth weight and its influence on neonatal mortality.

	Birth Weight	Birth Rate in Spain (2022)	Probability of Dying in the Neonatal Period
Term newborn	≥2.500 kg	97.86%	1×
Low birth weight	2.499–1.500 kg	6.61%	40×
Very low birth weight	<1.500 kg	0.82%	200

**Table 3:** Developmental alteration in intestinal drug absorption in neonates.

Physiological Factors	State
Gastric pH	>5
Gastric emptying time	Irregular
Gut motility	Reduced
Intestinal surface	Reduced
Microbial colonization	Reduced
Biliary function	Immature

**Table 4:** Characteristics of available vascular catheter types.

Catheter Type	Application	Characteristics	Issues
Peripheral venous catheter	Most intravenous medications, isotonic intravenous fluids, and blood transfusion	Low flow rates; physicochemical irritation with some medications produces phlebitis	Difficult to insert in newborns due to small, difficult to visualize vessels
Umbilical venous catheter	For diagnostic and therapeutic purposes: drug infusion, total parental nutrition, hypertonic intravenous fluids, central venous pressure, and venous blood gas monitoring and blood transfusions	It is usually placed within 12 h after birth, if indicated, for parenteral nutrition and/or inotropic support	Suitable only for newborns, as the umbilical vein remains for up to two weeks after birth
Peripherally inserted central catheter	Administration of intravenous medications and fluids, total parenteral nutrition, and blood sampling	Suitable for irritating and hyperosmolar drugs; together with the umbilical venous catheter, it helps reduce the risk of drug incompatibilities	Not suitable for the administration of large volumes in emergency situations
